# Diversity in Academic Otolaryngology: Career Outcomes for Underrepresented Groups

**DOI:** 10.7759/cureus.109389

**Published:** 2026-05-21

**Authors:** Lisa Velez-Velez, Areeb Y Shah, Gabriella Boone, Matthew C Simpson, Thomas Sanford

**Affiliations:** 1 Department of Otolaryngology - Head and Neck Surgery, Saint Louis University School of Medicine, St. Louis, USA; 2 Advanced HEAlth Data (AHEAD) Institute, Saint Louis University, St. Louis, USA

**Keywords:** academic otolaryngology, diversity, equity, physician workforce disparities, underrepresented in medicine

## Abstract

Objective

The aim of this study was to evaluate disparities in gender and racial/ethnic representation among otolaryngology faculty and assess how these demographics correspond with academic rank, research productivity, and institutional diversity initiatives.

Methods

This is a cross-sectional analysis. Faculty from 102 otolaryngology residency programs were reviewed between May and June 2024 using public registries. Data included demographics, academic title, leadership roles, and research productivity. Generalized estimating equations were employed to assess associations between race/ethnicity, gender, and career outcomes, adjusting for confounders.

Results

Of the 1,802 identified faculty, 118 (6.6%) identified as underrepresented in medicine and 536 (29.8%) were female. Faculty underrepresented in medicine demonstrated fewer citations (adjusted mean ratio [AMR] = 0.68, 95% confidence interval [CI] 0.49-0.93), but other productivity and advancement metrics were comparable to non-underrepresented peers. Female faculty had fewer years in practice (AMR = 0.65, 95% CI 0.58-0.72), lower h-index (AMR = 0.76, 95% CI 0.69-0.85), fewer publications (AMR = 0.64, 95% CI 0.54-0.76), and fewer citations (AMR = 0.69, 95% CI 0.54-0.87). Institutions with diversity, equity, and inclusion initiatives demonstrated increased female-to-male faculty ratios (adjusted odds ratio [AOR] = 1.33, 95% CI 1.07-1.65), though no association was observed for those identified as underrepresented in medicine.

Conclusion

While faculty underrepresented in medicine remain few in academic otolaryngology, those in faculty positions achieve comparable career outcomes. Female otolaryngologists experience disparities in productivity and academic advancement. Diversity, equity, and inclusion initiatives are associated with greater gender parity but require targeted expansion to address racial/ethnic advancement.

## Introduction

As the U.S. population becomes more diverse, diversity within the physician workforce remains an important topic. While individuals identifying as White alone currently comprise the majority of the population, minority racial groups such as Hispanic/Latino, Black, and Asian have grown significantly over the past decade and are projected to become the majority population over the next few decades [1]. Gender demographics in the United States are expected to remain stable, with slightly over 50% of the population being female [2]. The demographic makeup of attending physicians, residents, and medical students differs from that of the general population. More physicians are White than any other race. Asian individuals make up a larger share of physicians compared to the general population, while Black, Hispanic/Latino, and female physicians are underrepresented [3]. The representation of women and minorities is even lower among academic faculty, and these disparities worsen with each subsequent level of academic rank [4]. This is especially apparent in surgical subspecialties, where women and minorities are further underrepresented [5].

The demographic differences between the general population and the physician workforce reflect barriers to women and minorities entering medicine and surgical subspecialties, as well as to achieving academic rank. There are many factors that may contribute, including a lack of mentors, increased discrimination, implicit biases, and incidents of harassment [6]. Studies show that patients, particularly minorities, experience higher satisfaction when their doctor shares their race or ethnicity [7]. This is especially crucial for minority populations, who face worse health outcomes compared to the general population [8]. At the academic level, a diverse faculty can broaden perspectives on research and teaching, strengthen mentorship, and ultimately improve patient care [9].

These disparities have not gone unnoticed. Over the past several years, the trends in diversity within otolaryngology have been published. For example, otolaryngology lags behind other surgical specialties in the representation of minorities and women among applicants, residents, and faculty. A study found that there is only 1% Black representation among otolaryngology professors in 2018, and this disparity in achieving full professorship exists most prominently in otolaryngology [10]. Additional longitudinal analysis of the otolaryngology workforce has demonstrated that although the proportion of women faculty increased from 21% to 37% from 2000 to 2020, parity across academic rank has not been achieved. Improvements in rank equity among Black and Hispanic/Latino faculty have also been reported but appear to be disproportionately concentrated among men [11]. Furthermore, trends in disparity are observed across various otolaryngology fellowships. It was found that White men and women had an advantage in the otolaryngology-head and neck surgery (OHNS) match, while several racial, ethnic, and gender minorities were disadvantaged [12].

While prior studies have demonstrated persistent disparities in otolaryngology workforce representation, limited data exist regarding the academic career outcomes of underrepresented faculty already practicing within academic institutions. The primary objective of this study was to characterize demographic representation, academic rank, leadership positions, and research productivity among faculty in U.S. otolaryngology residency programs. A secondary objective was to evaluate associations between race/ethnicity, gender, and institutional diversity initiatives.

## Materials and methods

Study design

This cross-sectional study used data obtained from publicly available registries and was considered exempt from the Saint Louis University Institutional Review Board. Using the American Medical Association FREIDA database, United States allopathic and osteopathic otolaryngology residency programs were identified. Military-based programs (n = 6) were excluded due to differences in faculty appointment structures, rank designation systems, and publicly available institution information compared with civilian programs. Programs that did not list their faculty or did not include academic appointments/titles on their websites were also excluded (n = 23). Faculty members with the titles assistant professor, associate professor, or professor were included in the study. Individuals were excluded if they were adjunct, part-time, emeritus faculty, or clinical instructors, or if they had no Scopus profile. Faculty lists were independently reviewed by members of the research team to confirm their eligibility and avoid duplicate inclusion across institutional websites. Data collection was performed for 102 programs and completed in May of 2024.

Data collection

Each program website was accessed to collect data on otolaryngology faculty members, including demographics, educational backgrounds, and program leadership positions. If not available in faculty biographies on program websites, data were searched for on Doximity, LinkedIn, Castle Connolly, and private-practice websites. Relevant data included sex, race/ethnicity, medical degree, advanced degrees, subspecialty fellowship training, academic title, last year of training, and program leadership. Because demographic information was not universally self-reported, categorization was intended to estimate workforce representation trends rather than define individual identity. Years in practice were calculated by subtracting the last year of training from 2023. Data on h-index, number of publications, and number of citations were obtained via Scopus author search. When multiple Scopus profiles were identified for a faculty member, institutional affiliation, specialty, and publication history were cross-referenced to confirm the correct author profile. Editorial board membership was determined using website listings for the following journals: Otolaryngology-Head and Neck Surgery, OTO Open, JAMA Otolaryngology-Head & Neck Surgery, Laryngoscope, Head & Neck, Annals of Otology, Rhinology & Laryngology, Laryngoscope Investigative Otolaryngology, International Forum of Allergy & Rhinology, Otology & Neurotology, and American Journal of Rhinology & Allergy. Journals were selected based on their relevance to core otolaryngology practice, subspecialty representation, and academic prominence within the field. The intent was to capture editorial leadership within journals most closely aligned with the specialty rather than to provide an exhaustive inventory of all possible editorial appointments. Due to variability in reporting and accessibility of editorial board rosters, editorial memberships outside of the selected journals may not have been captured. Each program website was also searched for content related to diversity, equity, and inclusion (DEI) initiatives. Programs were classified as having DEI initiatives if departmental or residency-specific webpages included dedicated diversity statements, committees, pathway programs, mentorship initiatives, or recruitment efforts explicitly related to diversity, equity, and/or inclusion.

Race/ethnicity categorization

Race/ethnicity were categorized by U.S. Census Bureau designations and assigned at the discretion of data collectors based on public photographs of each individual, biographical data on public websites, professional profiles, pronoun usage, and geographical prevalence of surnames. When available, self-identified demographic information was used preferentially. In cases where demographic information was not explicitly stated, categorization was inferred based on publicly available materials. To reduce misclassification bias, any uncertainty regarding race/ethnicity categorization of a certain individual was resolved by independently cross-checking between two or three members of the research team. If a conclusion could not be reached, the individual was excluded from the analysis. Faculty members were categorized as underrepresented in medicine (URM) versus non-URM based on the Association of American Medical Colleges definition. Non-URM groups included White and Asian, whereas URM race/ethnicity categories included American Indian or Alaska Native; Black or African American; Hispanic, Latino, or of Spanish Origin; or Native Hawaiian or Other Pacific Islander.

Statistical analysis

Provider and program characteristics were summarized using counts and percentages or means and standard deviations (SDs) as appropriate. Variables included in adjusted models were selected before analysis was performed based on prior literature and theoretical relevance to academic productivity and advancement. Generalized estimating equations (GEEs) estimated the association of relevant provider-level and program/division-level characteristics with career outcomes while accounting for program/division-level clustering. The GEEs for mean years in practice, h-index, number of publications, and number of citations used a Poisson distribution with a natural logarithm link; the GEEs for having multiple advanced degrees, having a program leadership position, and being a journal editor used a binomial distribution with a logit link; and the GEE for academic title used a multinomial distribution with a generalized logit link. GEEs using a binomial distribution with a logit link also estimated the association of program/division-level characteristics with URM to non-URM ratio and female-to-male ratio. For the GEEs with the outcomes of academic title, leadership position, URM to non-URM ratio, and female-to-male ratio, providers/programs/divisions from Puerto Rico were not included because only one institution in Puerto Rico was included in the study. Poisson results are reported as crude mean ratios (CMRs) and adjusted mean ratios (AMRs) with 95% confidence intervals (CIs), and binomial and multinomial results are reported as crude odds ratios (CORs) and adjusted odds ratios (AORs) with 95% CIs. Analysis was performed using SAS version 9.4 (SAS Institute, Cary, NC). All tests were two-tailed with α = 0.05.

## Results

Provider characteristics

This study included 1,802 providers from 102 programs, although one provider’s years in practice could not be determined and was only included in program-level results. Only 118 (6.6%) of providers were from URM race/ethnicity groups, with 70 (3.9%) of providers being Hispanic/Latina/Latino and 48 (2.7%) of providers being Non-Hispanic Black. Slightly less than one-third, 536 (29.8%) of providers were female. Of the URM providers, only 44 (37.3%) were female. The largest group of academic otolaryngology providers practiced at institutions in the South (610 [33.9%]), followed by the East (482 [26.8%]), Midwest (370 [20.5%]), West (326 [18.1%]), and Puerto Rico (13 [0.7%]). Nearly all providers held an MD (1,787 [99.2%]), and a majority completed at least one fellowship (1,529 [84.9%]). About one-fifth (327 [18.2%]) providers completed at least one additional advanced degree. Most providers were assistant professors (712 [39.5%]), followed by associate professors (547 [30.4%]) and professors (542 [30.1%]), and 231 (12.8%) held at least one leadership position within their department or division. The average number of years in practice was 13.9 (SD 10.8). The average h-index was 15.5 (SD 13.5), the average number of publications was 57.3 (SD 72.4), and the average number of citations was 1,687.2 (SD 4,418.9). In addition, 256 (14.2%) of providers held editorial positions for academic journals (Tables 1, 2).

**Table 1:** Characteristics of academic otolaryngologists in the United States and Puerto Rico as of 2023 DEI, diversity, equity, and inclusion; SD, standard deviation; URM, underrepresented in medicine

Provider Level Characteristics	n (%) or Mean (SD), n = 1,801
URM - No	1,683 (93.4%)
URM - Yes	118 (6.6%)
Asian	394 (21.9%)
Black	48 (2.7%)
Hispanic/Latina/Latino	70 (3.9%)
White	1,289 (71.6%)
Female	536 (29.8%)
Male	1,265 (70.2%)
Years in practice	13.9 (10.8)
Region - East	482 (26.8%)
Region - Midwest	370 (20.5%)
Region - Puerto Rico	13 (0.7%)
Region - South	610 (33.9%)
Region - West	326 (18.1%)
Program/department-specific DEI initiative or language - No	1,106 (61.4%)
Program/department-specific DEI initiative or language - Yes	695 (38.6%)
DO	9 (0.5%)
MBBCh	2 (0.1%)
MBBS	3 (0.2%)
MD	1,787 (99.2%)
Fellowship - No	272 (15.1%)
Fellowship - Yes	1,529 (84.9%)
Other advanced degree - No	1,474 (81.8%)
Other advanced degree - Yes	327 (18.2%)
Assistant professor	712 (39.5%)
Associate professor	547 (30.4%)
Professor	542 (30.1%)
Leadership position - No	1,570 (87.2%)
Leadership position - Yes	231 (12.8%)
h-index	15.5 (13.5)
Number of publications	57.3 (72.4)
Number of citations	1,687.2 (4,418.9)
Journal editor - No	1,545 (85.8%)
Journal editor - Yes	256 (14.2%)

**Table 2:** Characteristics of academic otolaryngology programs in the United States and Puerto Rico as of 2023 DEI, diversity, equity, and inclusion; SD, standard deviation

Program Characteristics	n (%), n = 102
Region - East	27 (26.5%)
Region - Midwest	22 (21.6%)
Region - Puerto Rico	1 (1.0%)
Region - South	35 (34.3%)
Region - West	17 (16.7%)
Program/department-specific DEI initiative or language - No	63 (61.8%)
Program/department-specific DEI initiative or language - Yes	39 (38.2%)

Career outcomes

Compared to non-URM providers, those from URM backgrounds had a significantly lower number of citations (AMR = 0.68, 95% CI 0.49-0.93) but were not significantly different from non-URM providers for any other career outcome after adjusting for potential confounders (Tables 3, 4). Compared to male providers, female providers had a significantly lower number of years in practice (AMR = 0.65, 95% CI 0.58-0.72), h-index (AMR = 0.76, 95% CI 0.69-0.85), number of publications (AMR = 0.64, 95% CI 0.54-0.76), and number of citations (AMR = 0.69, 95% CI 0.54-0.87) after adjusting for potential confounders. Before adjusting for potential confounders, the associate professor to assistant professor ratio and the professor to associate professor ratio were significantly lower for females than males (associate to assistant COR = 0.61, 95% CI 0.46-0.80; professor to associate COR = 0.47, 95% CI 0.36-0.62). However, after adjusting for potential confounders, comparisons by sex were no longer significant (associate to assistant AOR = 0.97, 95% CI 0.73-1.28; professor to associate AOR = 1.12, 95% CI 0.77-1.63) (Table 5). The attenuation of the association between sex and academic title appears to be independently attributable to both years in practice and number of publications. When only adjusting for years in practice in the association between sex and title, the estimated association between sex and title was smaller (associate to assistant AOR = 0.76; professor to associate AOR = 0.72). Similar results are found when only adjusting for number of publications in the association between sex and title (associate to assistant AOR = 0.73; professor to associate AOR = 0.77). However, when adjusting for years in practice and number of publications together, the estimates change more than in either of the previous estimates, with the associate to assistant AOR being close to 1 (0.96) and the professor to associate AOR being non-significantly higher for females than for males (AOR = 1.12) (Table 6).

**Table 3:** Comparisons of career outcomes between URM and non-URM academic otolaryngologists. ^1^Adjusted for URM and sex. ^2^Adjusted for URM, sex, and years in practice. ^3^Adjusted for URM, sex, years in practice, and number of publications. URM, underrepresented in medicine

Variables	Non-URM, n = 1,683 (93.4%)	URM, n = 118 (6.6%)	URM vs Non-URM	
Years in practice	14.0 (10.9)	13.0 (9.5)	0.93 (0.82-1.05)	0.96 (0.84-1.09)^1 ^
h-index	15.8 (13.5)	11.4 (11.9)	0.72 (0.54-0.96)	0.78 (0.58-1.03)^2 ^
Number of publications	58.3 (71.6)	42.5 (81.0)	0.73 (0.49-1.08)	0.80 (0.54-1.18)^2 ^
Number of citations	1,732.5 (4,509.4)	1,040.4 (2,761.3)	0.60 (0.36-1.01)	0.68 (0.49-0.93)^3 ^

**Table 4:** Comparisons of career outcomes between URM and non-URM academic otolaryngologists, continued. ^*^Results for academic title and leadership position do not include providers practicing in Puerto Rico. ^1^Adjusted for URM and sex. ^2^Adjusted for URM, sex, and years in practice. ^3^Adjusted for URM, sex, years in practice, and number of publications. ^4^Adjusted for URM, sex, years in practice, number of publications, and program region. ^5^Adjusted for URM, sex, years in practice, program region, and academic title. AMR, adjusted mean ratio; AOR, adjusted odds ratio; CI, confidence interval; CMR, crude mean ratio; COR, crude odds ratio; SD, standard deviation; URM, underrepresented in medicine

Career Outcomes	Non-URM n (Column %), n = 1,683 (93.4%)	URM n (Column %), n = 118 (6.6%)	URM vs Non-URM COR (95% CI)	URM vs Non-URM AOR (95% CI)
Other advanced degree - No	1,377 (81.8%)	97 (82.2%)	Reference	Reference
Other advanced degree - Yes	306 (18.2%)	21 (17.8%)	0.97 (0.59-1.61)	0.97 (0.59-1.61)^2^
Assistant professor^*^	651 (38.7%)	51 (48.6%)	Reference	Reference
Associate professor^*^	521 (31.0%)	25 (23.8%)	Reference = Assistant 0.61 (0.38-0.98)	Reference = Assistant 0.62 (0.36-1.08)^4^
Professor*	511 (30.4%)	29 (27.6%)	Reference = Associate 1.18 (0.73-1.91)	Reference = Associate 1.72 (0.93-3.18)^4^
Leadership position - No*	1,466 (87.1%)	92 (87.6%)	Reference	Reference
Leadership position -Yes*	217 (12.9%)	13 (12.4%)	0.95 (0.56-1.64)	1.02 (0.56-1.85)^5^
Journal editor - No	1,438 (85.4%)	107 (90.7%)	Reference	Reference
Journal editor - Yes	245 (14.6%)	11 (9.3%)	0.60 (0.34-1.07)	0.72 (0.40-1.30)^3^

**Table 5:** Comparisons of career outcomes between female and male academic otolaryngologists. ^1^Adjusted for URM and sex. ^2^Adjusted for URM, sex, and years in practice. ^3^Adjusted for URM, sex, years in practice, and number of publications. AMR, adjusted mean ratio; AOR, adjusted odds ratio; CI, confidence interval; CMR, crude mean ratio; COR, crude odds ratio; SD, standard deviation; URM, underrepresented in medicine

Variables	Male, Mean (SD), n = 1,265 (70.2%)	Female, Mean (SD), n = 536 (29.8%)	Female vs Male CMR (95% CI)	Female vs Male AMR (95% CI)
Years in practice	15.6 (11.4)	10.0 (8.3)	0.64 (0.58-0.72)	0.65 (0.58-0.72)^1 ^
h-index	17.5 (14.6)	10.7 (8.8)	0.61 (0.53-0.71)	0.76 (0.69-0.85)^2^
Number of publications	67.3 (80.9)	33.8 (37.1)	0.50 (0.41-0.62)	0.64 (0.54-0.76)^2 ^
Number of citations	2,073.4 (5,121.5)	775.7 (1,594.6)	0.37 (0.26-0.54)	0.69 (0.54-0.87)^3 ^

**Table 6:** Comparisons of career outcomes between female and male academic otolaryngologists, continued. ^*^Results for academic title and leadership position do not include providers practicing in Puerto Rico. ^1^Adjusted for URM and sex. ^2^Adjusted for URM, sex, and years in practice. ^3^Adjusted for URM, sex, years in practice, and number of publications. ^4^Adjusted for URM, sex, years in practice, number of publications, and program region. ^5^Adjusted for URM, sex, years in practice, program region, and academic title. AMR, adjusted mean ratio; CMR, crude mean ratio; URM, underrepresented in medicine

Variables	Male, n (Column %), n = 1,265 (70.2%)	Female, n (Column %), n = 536 (29.8%)	Female vs Male CMR (95% CI)	Female vs Male AMR (95% CI)
Other advanced degree - No	1,035 (81.8%)	439 (81.9%)	Reference	Reference
Other advanced degree - Yes	230 (18.2%)	97 (18.1%)	0.99 (0.74-1.33)	0.99 (0.73-1.36)^2 ^
Assistant professor^*^	417 (33.2%)	285 (53.5%)	Reference	Reference
Associate professor^* ^	386 (30.8%)	160 (30.0%)	Reference = assistant 0.61 (0.46-0.80)	Reference = Assistant 0.97 (0.73-1.28)^4 ^
Professor*	452 (36.0%)	88 (16.5%)	Reference = associate 0.47 (0.36-0.62)	Reference = associate 1.12 (0.77-1.63)^4 ^
Leadership position - No^*^	1,085 (86.5%)	473 (88.7%)	Reference	Reference
Leadership position - Yes^* ^	170 (13.5%)	60 (11.3%)	0.81 (0.59-1.12)	1.10 (0.77-1.57)^5 ^
Journal editor - No	1,075 (85.0%)	470 (87.7%)	Reference	Reference
Journal editor - Yes	190 (15.0%)	66 (12.3%)	0.79 (0.58-1.09)	1.38 (0.93-2.06)^3 ^

Program characteristics

Neither region nor whether a program had a program/division-specific DEI initiative or language was associated with URM to non-URM ratio. However, programs with DEI initiatives or language had a female-to-male ratio that was 1.33 times higher than programs without DEI (AOR = 1.33, 95% CI 1.07-1.65) (Table 7).

**Table 7:** Comparison of association estimates for sex (female versus male) with academic title. AOR, adjusted odds ratio; CI, confidence interval; COR, crude odds ratio

Adjusted for	Associate-to- Assistant Ratio COR/AOR (95% CI)	Associate-to-Assistant Ratio Percentage Change From Crude	Professor-to-Associate Ratio COR/AOR (95% CI)	Professor-to-Associate Ratio Percentage Change From Crude
Crude estimate	0.61 (0.46-0.80)	Not applicable	0.47 (0.36-0.62)	Not applicable
Years in practice	0.76 (0.57-1.01)	25.0	0.72 (0.53-0.99)	54.1
Number of publications	0.73 (0.55-0.97)	21.1	0.77 (0.57-1.04)	63.2
Years in practice and number of publications	0.96 (0.72-1.29)	59.0	1.12 (0.77-1.62)	138.1

## Discussion

Otolaryngology remains among the least diverse surgical fields. According to the 2023 U.S. Physician Workforce Data Dashboard report, out of 10,178 active academic and non-academic otolaryngologists in the U.S., 2.4% were Black or African American and 4.1% were Hispanic or Latino [13]. Our study found similar representation with URM faculty representing only 118 (6.6%) of faculty. As our field continues to highlight the importance of diversifying the otolaryngology workforce, analyzing the current cohort of URM faculty and their academic career outcomes can help inform initiatives to improve representation. This study examines diversity among academic otolaryngologists nationwide, comparing qualifications and career trajectories across demographic characteristics. The current gap in racial and ethnic diversity within academic otolaryngology, along with the continued gender imbalance with only 536 (28.9%) female faculty identified in our study, raises concerns about systemic barriers in recruitment, retention, and career advancement [14]. Among URM providers, a disproportionate number were male, with only 44 (37.3%) being female, meaning that of all providers, only three (2.4%) were both URM and female. This highlights the significance of intersectionality. Those who belong to more than one underrepresented group may face compounding challenges that are not captured when examining each identity in isolation.

Impact of demographics on career outcomes

The lack of diversity in academic otolaryngology may lead to disparities in career outcomes based on demographic characteristics. It is known that racial and gender minorities often face reduced opportunities for career and rank advancement compared to the general academic physician population [15]. Our data indicates that URM providers have significantly fewer citations compared to Non-URM providers, suggesting visibility barriers; however, other metrics such as publications, h-index, and years in practice are similar to non-URM counterparts, indicating URM providers produce high-quality research despite facing visibility challenges.

Our data show that female academic otolaryngologists have fewer years in practice, a lower h-index, fewer publications, and fewer citations than their male colleagues. Female providers across specialties face well-documented challenges with advancement and productivity, and our data show that this is also a concern in academic otolaryngology [16]. Similar findings have been reported internationally. A study of Canadian academic otolaryngologists demonstrated that men had higher overall publication metrics, including h-index and senior authorship; however, gender disparities were not observed in early career stages, suggesting differences may emerge over time [17]. In our initial analysis, female providers had a lower academic rank than males on average; however, after adjusting for confounders such as years in practice and number of publications, these differences were no longer significant. This suggests that differences in academic rank are mediated by years in practice and research productivity. Some data, including findings within otolaryngology specifically, suggest that work-home conflicts and additional responsibilities, such as childcare, faced by female providers may limit their research time and contribute to lower academic rank [18,19]. These findings may reflect a persistent gender disparity in academic and research achievement.

Role and limitations of DEI initiatives

We also explored the impact of program specific DEI initiatives on diversity metrics. Despite increasing DEI efforts, gender and racial disparities persist [20]. Our data found that while DEI initiatives were linked to increased gender diversity, they did not impact racial or ethnic diversity. While DEI efforts may be improving gender representation, they may be less effective in addressing the challenges faced by racial and ethnic minorities. It should be noted that the presence or absence of DEI language on currently available program websites may not fully reflect institutional priorities or historical initiatives, given the current political climate. Regardless, targeted interventions prioritizing inclusion, advancement, and recruitment are needed to address URM disparities.

Mentorship, systemic barriers, and the value of a diverse workforce

The lack of diversity starts upstream in academic training. Previous reports have shown that URM groups are underrepresented in medical school and remain so as they progress along the academic pipeline [10]. This underrepresentation can be attributed to several barriers, including the lack of mentorship, discrimination, implicit biases, and harassment, which are particularly pronounced in surgical subspecialties. Although over half of U.S medical school graduates are female and 20% identify as URM, our data indicate that only 536 (30%) of academic otolaryngologists are women and just 118 (6.6%) are URM [13]. Lower recruitment of these populations is compounded by factors that deter them from pursuing a surgical career, including the perception of increased sex- and race-based discrimination, limited presence of similar peers and role models, and concerns related to family planning [21]. Structural barriers in recruitment and hiring, including institutional practices and implicit bias in faculty selection, may also contribute to underrepresentation in the hiring process itself. Once within the academic environment, these groups face barriers that impair long-term retention, including higher rates of microaggressions, mistreatment, and isolation, which are all known contributors to burnout and attrition [22]. Reduced access to research support and funding may contribute to differences in scholarly productivity [23]. Importantly, these barriers are systemic and extend beyond any individual institution. Further research is needed to clarify how these structural barriers shape diversity within academic otolaryngology.

Our data also show that only three (2.4%) academic otolaryngologists are both URM and female, suggesting limited access to identity-concordant mentorship [24]. Structured mentorship has been shown to improve URM faculty promotion and leadership outcomes, while women and URM faculty often face reduced access to high-quality mentorship and sponsorship, exacerbating disparities in academic progression [25]. Our data indicate that URM providers had significantly fewer citations compared to non-URM providers, which may reflect differences in research visibility, mentorship access, institutional support, or professional network effect. [24].

Furthermore, a diverse workforce leads to higher patient satisfaction, as patients are more likely to feel understood and comfortable when treated by providers with similar backgrounds, resulting in better care and health outcomes [26,27]. Reduced access to research support and funding may contribute to differences in scholarly productivity [27]. In academic environments, a diverse faculty enhances mentorship and fosters a more inclusive atmosphere, which is vital for the development of future healthcare leaders [28]. These benefits are particularly relevant to academic otolaryngology, where underrepresentation of URM and female providers could affect the quality of patient care and limit the scope of research and teaching. Given that academic otolaryngologists serve as the primary role models for trainees who will become the leaders of our field, diversity and representation among faculty members in residency programs are likely to play a key role in addressing current disparities.

Future directions

Several strategies must be implemented to improve the representation of URM and women in academic otolaryngology. Enhancing mentorship programs is crucial, particularly those that connect these providers with mentors who can guide them through their unique challenges. Such programs should be designed to provide academic guidance, career development, and networking opportunities. Addressing discrimination and implicit biases through targeted training and policies is essential to creating an inclusive environment that supports the advancement of all individuals. Creating more practical DEI initiatives will also be key to sustaining progress, though it will be challenged by the current political environment. Future research should explore the career pathways of minority academics in greater depth and investigate the long-term impact of DEI initiatives on career outcomes and institutional culture. Such research will be critical in developing evidence-based strategies to foster a more diverse and inclusive academic environment in otolaryngology.

Importantly, this analysis was conducted using data collected prior to recent changes in the national policy environment surrounding DEI initiatives. Ongoing shifts in institutional priorities and external regulations may influence the implementation of DEI efforts and, consequently, the future composition and career trajectories within academic otolaryngology.

Limitations

The cross-sectional design limits our ability to establish causality between demographic characteristics and career outcomes. Additionally, reliance on publicly available data may not capture nuances such as informal mentorship or unreported discrimination. Publicly available institutional websites may not fully reflect current faculty composition, leadership appointments, or evolving departmental initiatives. Selection bias may also exist, as programs without publicly available faculty information or academic appointment data were excluded from analysis. The authors acknowledge that assigning race/ethnicity and sex using publicly available data may introduce misclassification and does not capture the full complexity of individual identity. Additionally, because demographic identity was not self-reported, this approach may result in an undercounting of individuals who self-identify as URM. However, this methodology has been used in prior workforce analyses when self-reported data are unavailable [29,30]. While we adjusted for several potential confounders, there may still be unmeasured variables that could influence the observed associations, such as personal choices regarding work-life balance. This study could not account for protected research time, institutional funding support, parental leave policies, clinical workload, or individual career preferences, all of which may influence academic productivity and advancement. Furthermore, the study could not fully assess intersectionality, including overlapping effects of race, gender, and socioeconomic status. This nuance is crucial for understanding the full spectrum of barriers faced by URM and female providers; however, our study was not equipped to analyze these intersecting factors in depth. Additionally, the exclusion of providers from Puerto Rico, due to its small sample size, limits the generalizability of our findings across regions. Future studies should incorporate longitudinal data and examine institutional and cultural factors.

## Conclusions

This study shows that disparities in representation within academic otolaryngology persist. Female physicians had lower research output, which appears to contribute to differences in academic advancement, though this likely reflects a combination of structural and institutional factors. In contrast, URM faculty demonstrated publication rates similar to their peers' but received fewer citations, suggesting that visibility and recognition of their work may differ despite comparable productivity. Taken together, these findings point toward ongoing barriers that are not fully addressed by current efforts. Improving access to mentorship, strengthening research support, and ensuring more equitable recognition of scholarly contributions will be important moving forward. These changes are especially relevant as the field continues to serve an increasingly diverse patient population, where a more representative workforce may better support both care and innovation.
